# Digital technology application on corporate internationalization: Co-moderating effect of centralizing decision power and internationalization experience

**DOI:** 10.1371/journal.pone.0306696

**Published:** 2024-10-17

**Authors:** Chunhao Li, Jiaqi Yin, Shengxiao Li, Hongyong Zhou

**Affiliations:** 1 Business School, Shaoxing University, Shaoxing, China; 2 Postdoctoral, Zhongnan University of Economics and Law, Wuhan, China; 3 Zhejiang Industry Polytechnic College, Shaoxing, China; University of Central Punjab, PAKISTAN

## Abstract

Digital technologies are a significant additional powerhouse for corporate internationalization and competitiveness. The existing research has not effectively distinguished the breadth and depth of internationalization, and there are also inadequate context considerations of how much digital technology applications influence enterprise internationalization. To bridge this gap, this paper has selected 203 valid questionnaires from manufacturing enterprises in Zhejiang Province, China, then elaborates and constructs a theoretical model based on existing studies, and using multiple linear regression analysis, finally to empirically assess and further explain the mechanisms of digital technologies’ influence on corporate internationalization. The results reveal that utilizing digital technologies has positive influence on both internationalization breadth and depth. Moreover, centralizing decision power negatively moderates the relationship between enterprises’ digital applications and their internationalization breadth (or depth), while internationalization experience has opposite influence, with these two factors jointly moderating such relationship. Finally, this research can contribute to better understanding of leveraging digital technologies to upgrade corporate internationalization and provides reasonable theoretical insights for enhancing deeper and more diversified enterprise internationalization.

## 1. Introduction

Since China’s accession to the WTO, Chinese enterprises have more active presence in the global market. However, they face great challenges such as economic crisis, escalating trade protectionism, the COVID-19 pandemic shock, and volatile international market demand [1]. As a result, the systematic and coherent internationalization has become a great concern for industries, the academia, and decision-makers alike.

To solve it, emerging digital and intelligent methods are growing to drive corporate internationalization. Many enterprises utilize digital technologies, such as cross-border e-commerce platforms, big data system for global customer analysis, and global Internet of Things system, to engage in worldwide activities and global competitive advantages, to gain more international presence [2]. According to previous studies, digitalization significantly influences international business, including export orientation, export quality, and export technological complexity [3–5], cross-border M&A [6], international competitive advantages [7], internationalization tendency [8], internationalization pace [2], internationalization degree [9–11], and internationalization performance [12, 13]. However, some scholars argue that merely digital technology application by enterprises (EDT) is not an effective solution to institutional and cultural differences, economic gap [14] or subsequent data security and data sovereignty [10]. Moreover, limited capabilities and excessive investments may lead to the “digitalization paradox” [15, 16], together with an inverted U-shaped relationship between digitalization and enterprise performance [17], which is not conducive to international expansion [18]. Meanwhile, digitalization indeed speeds up international expansion, but there are limitations such as product diversification, boundary conditions [19], and regional heterogeneity [20]. In this aspect, existing research has emphasized the positive influence of digital technology application on enterprise internationalization and explored its negative effects, laying the foundation for this study.

Generally, internationalization can be measured by its breadth and depth. More specifically, the breadth refers to the diversity and complexity of an enterprise’s transaction operations, reflected by its presence in how many countries or regions. The depth refers to the proportion of overseas sales (or assets) to total sales (or total assets), indicating an enterprise’s dependency on overseas contributions [21]. It is clear that these two dimensions diverge, so separate discussions accordingly should be conducted. In particular, internal and external contextual factors of organizations may significantly influence EDT’s effect on corporate internationalization. Among them, decision power concentration (DC) reflects centralization level of decision power and authority of top leadership [22], which may result in lower decision quality and efficiency, or even decision errors and inadequate incentive effects, finally hindering knowledge sharing [23–26]. In addition, rich internationalization experience is precious to better understand the institutional and cultural attributes of host countries, international business regulations, international market knowledge, and the development of global innovation networks, to grasp internationalization timing and corresponding strategic orientation [27–29].

In summary, existing research has following shortcomings. The first is inadequate in-depth explorations of EDT’s negative effects [30] and next is insufficient attention paid to the breadth and depth of internationalization, which needs to be distinguished and studied due to their essential differences [10, 21]. Thirdly, there are few context considerations of EDT’s influence on enterprise internationalization, and further exploration is needed on what influencing factors promote or hinder the effectiveness of EDT [30].

To bridge the above gaps, this study proposes the following questions. Does the EDT positively influence the enterprises internationalization? What are the differences in the results affected by the breadth and depth of internationalization? What are favorable conditions to further facilitate such influence?

To answer the above questions, this study needs to analyze two parts, one is the above-mentioned influence from breadth and depth dimensions, and the other is the separate moderating effects and co-moderating effect of DC and internationalization experience (IE). Based on data from manufacturing enterprises in Zhejiang Province, this study builds a theoretical model that verified by empirical analysis, and finally hypotheses are confirmed.

The possible theoretical contributions of this study include the following aspects. (1) Explaining and empirically verifying EDT’s influence on the breadth and depth of internationalization, thereby enriching digitalization materials in international business. (2) Revealing the different influence of DC and IE and expanding the boundaries of how EDT affects internationalization breadth and depth, thus addressing the limitations of external exploration in understanding the mechanisms of EDT. In addition, the potential pragmatic significance of this study is to provide a theoretical foundation for enterprises to utilize digital technologies for international expansion. That is, enterprises need to consider the differences of DC and IE when adopting different internationalization strategies, which can refer to practical inspirations in this paper for the rapid expansion of enterprise internationalization.

This study mainly consists of five parts, namely introduction, theoretical foundation and research hypotheses, sample and variable measurement, empirical analysis, and conclusions and implications.

## 2. Theoretical foundation and research hypotheses

### 2.1 Digital technology application and corporate internationalization

Firstly, digital technology application is useful to identify international market opportunities. Advanced tracking techniques can be adopted to monitor, record, and understand consumer behaviors and preferences, thus rapidly grasping international market demand and further relevant knowledge [3]. Therefore, the EDT aids in efficient identification and selection of potential market opportunities and unique resources [31], to enhance enterprises’ assessment and forecast of international market demands [32] despite geographical and cultural barriers that slow corporate international expansion [33]. Additionally, through intelligent production and technological self-growth, enterprises can respond timely to international market demands, realize product and service iteration [34], and meet the increasingly differentiated consumers’ needs at lower costs through customization and service extension [35]. Furthermore, Internet platform is another tool to integrate personalized demands from international markets, thus realizing scale economies [36], and greater international opportunities.

Next, digital technology application is beneficial for international market development. By deep engagement in activities on cross-border digital platforms, such as intelligent warehouses and modularized production, enterprises can absorb heterogeneous knowledge, save resources for independent experiments, reduce product development time and risk, and speed up product innovations [7]. For example, Haier’s use of 3D printing to deliver personalized “Cabinet” refrigerators within 7 days has hugely strengthened its international competitiveness. In addition, digital technologies can facilitate real-time tracking, monitoring, and coordination, thus optimizing inter-organizational collaborations and internal business flows for higher production efficiency and better control over strategic resources, finally maintaining a consistent internationalization pace [37]. Beyond, by leveraging advanced manufacturing systems, automatic processes and tracking technologies, etc., enterprises can enhance flexibility and adaptability, and establish operational processes that align with international market requirements, so that they can cost-effectively allocate resources and respond to potential risks. What’s more, the programmability and reconfigurability of digital technologies can also be utilized for resource re-integration, to better fit the international market without organizational and situational limits [10].

Finally, stronger capability for digital application is conducive to upgrading product development process with higher proficiency, subsequently facilitating sales growth and market share rise of new products [38, 39]. Based on above discussions, the following hypotheses are proposed:

H1: Digital technology application positively influences the breadth of internationalization.
H2: Digital technology application positively influences the depth of internationalization.

### 2.2 The moderating effect of DC

Decision centralization reflects decision power and authority centralized to senior leaders [22], or specifically the allocation of power within an organization between top-level and lower-level managers, indicating the exclusive use or disposal of enterprise resources by senior management. Considering increasingly turbulent and uncertain international markets, DC is negative to further overseas expansion. To explain, low decision quality comes first. DC hinders the transmission of information and capabilities within the organization. Compared to grass-root managers, lower accuracy and timeliness of information transmission will result in senior managers’ insufficient understanding of international market demands and overseas environments. At the same time, DC leads to managers’ excessive focus on a certain field, so they are unable to fully share team knowledge, thus resulting in ineffective decision-making [40]. In that way, DC not only fails to promptly respond to complex and ever-changing international market demands, resulting in decision errors, investment opportunity losses, and reduce investment efficiency [25], but also may reinforce self-interest motives and behaviors among senior managers towards worse corporate performance [14] and international expansion. In this aspect, previous studies have already shown that DC distorts top-level investment decisions to excessively pursue projects with negative net present value, thus increasing the risk of stock price collapse [23]. Secondly, DC may lead to excessive resource concentration. Despite higher organizational efficiency [25], it faces high risks, information asymmetry, and other international market activities. Once DC fails, it will significantly [23] affect the diversification and deep expansion of the international market. Finally, DC is not beneficial to motive grass-root employees, so that they will have a stronger sense of distrust and risk aversion, finally leading to their reluctance to apply intellectual resources such as knowledge and skills to relevant enterprise activities [41], typically project investments, effective allocation of human resources, and recommendations for compensation policy designs. In a word, these employees’ motivations fail to be effectively stimulated [25], which cannot build a more vibrant grassroots team or consequently more active international activities. On the third place, DC is not favorable to inter-departmental cooperation by inhibiting horizontal communication and coordination among employees within the organization, thus hindering knowledge sharing, absorption, and transformation [42]. Faced with the variability and complexity of the international market-oriented environment, as well as the diversity and personalization of market demand, it is not conducive to optimal response strategies. In conclusion, excessive DC hinders the positive influence of digital technology application on enterprise internationalization together with enterprises’ participation in international competitions. Based on that, the following hypotheses are proposed:

H3: DC negatively moderates the relationship between digital technology application and the breadth of internationalization.
H4: DC negatively moderates the relationship between digital technology application and the depth of internationalization.

### 2.3 The moderating effect of IE

Rich IE is beneficial to be familiar with host countries’ institutions and cultural attributes, international business operation regulations, international market knowledge, and the building of international innovation networks [27, 28], further aiding in selecting internationalization timing and corresponding strategy orientation [29]. Specifically, enterprises with abundant IE can utilize existing experience and resources to analyze diverse overseas information. So that they can better identify precious opportunities for overseas expansion and recognize potential high risks by absorption and conversion, to avoid premature decisions and propose targeted strategic plans for final success [43, 44]. Moreover, liabilities of foreignness hinder knowledge spillover and increase coordination and control pressures, especially cultural and economic distance hinders innovation in cross-border digitalization [14]. Meanwhile, this also increases environmental uncertainty and blurs decision costs and probabilities [11]. But IE is powerful in better coordinating cultural and interest conflicts in international cooperation, overcoming the liabilities of foreignness [27, 43]. Extensive IE enables enterprises to familiarize with and adapt to the host country’s environment through available experience, then they can better acquire local information, technologies and business networks, further enhancing their organizational management capabilities in similar environments, to reduce uncertainty, mitigate the liabilities of foreignness, and reduce coordination costs [45, 46]. It is useful to deal with complex, dynamic, and diverse international environment, thus better exploring and adapting to new international markets. Enterprises with abundant IE are more likely to understand and grasp information regarding market environment changes of specific host countries [47], such as demand fluctuations and technological dynamics. In this way, these enterprises will better figure out and utilize opportunities and conduct legitimate operations in the local market, make prudent market expansion decisions, thereby reducing relevant risks of international investment. Research has shown that international diversification experience can buffer the adverse effects of internal resources on international expansion [18], while high-level internationalization experience can increase the performance benefits brought by digitalization [17]. This study holds that IE can manifest an enterprise’s ability and helps alleviate costs associated with digital technology investment, such as knowledge gathering and communication coordination. Additionally, it strengthens the identification, learning, and utilization of international information, knowledge, and opportunities during digital technology application, and together weakens the liabilities of foreignness, positively influencing the depth and breadth of corporate internationalization. Based on that, the following hypotheses are proposed:

H5: IE positively moderates the relationship between digital technology application and the breadth of internationalization.
H6: IE positively moderates the relationship between digital technology application and the depth of internationalization.

### 2.4 Co-moderating effect

DC and IE reflect the internal and external contexts of an organization separately. According to the mechanism of the relationship between digital technology application and corporate internationalization, the efficiency of resource allocation, resource integration effect, employee incentive effect, and reduction of agency problems required for the internationalization of enterprises formed by the DC are required [24, 25, 40]. However, excessive DC can reduce decision-making quality, speed, and organizational efficiency, which is not conducive to employee initiative and cross departmental cooperation [40, 25, 41, 42], and hinder the positive influence of EDT on enterprise internationalization. Similarly, it is also necessary to ensure the capabilities or resources that enterprises rely on for internationalization brought about by IE, in order to optimize their internationalization strategy choices, overcome the disadvantages of outsiders, and promote international expansion of enterprises [17, 29, 43]. That is, these two factors jointly influence the relationship between digital technology application and corporate internationalization. Based on hypotheses H3, H4, H5, and H6, DC has a negative moderating effect, while IE has an opposite effect. Therefore, lower decision power concentration and more internationalization experience are more conducive to enterprise internationalization. Considering the above analysis, the following hypotheses are proposed:

H7: DC and IE jointly moderate the relationship between digital technology application and the breadth of internationalization.
H8: DC and IE jointly moderate the relationship between digital technology application and the depth of internationalization.

**Fig 1:**
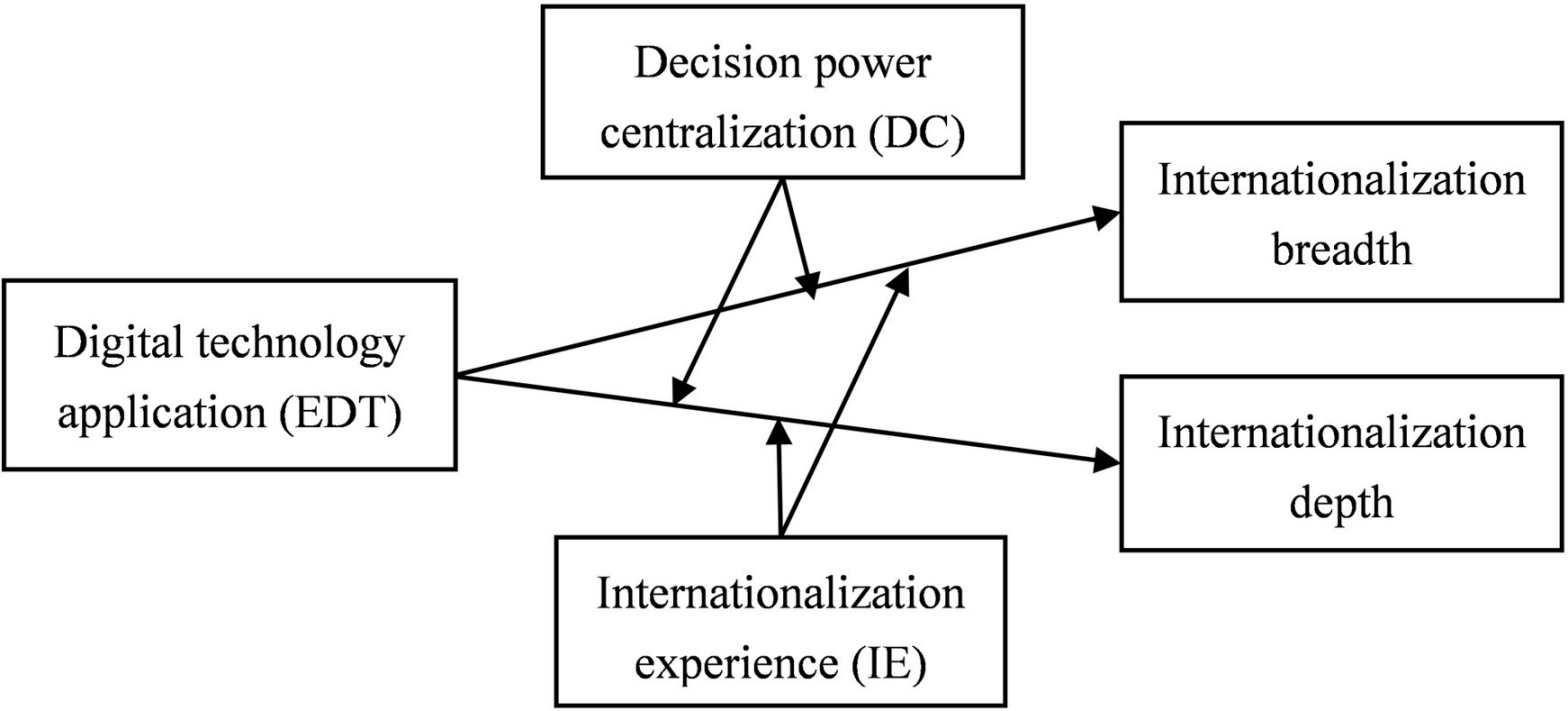
Conceptual model.

Based on the above analysis, we have built a conceptual model, as shown in Fig 1.

## 3. Sample and variable measurement

### 3.1 Research objectives

To fill the existing research gaps, this article identifies two main goals. Firstly, it has analyzed the influence of EDT on the breadth and depth of internationalization. Secondly, it has explored the separate moderating and co-moderating effects of DC and IE. Based on the research results, reasonable and effective suggestions are proposed to provide references for the internationalization and digital development of enterprises.

### 3.2 Research methods

To answer above questions, this study used a questionnaire survey method for data collection. This mature and standardized method is easy to implement and control, so it is widely used in social survey research, which is always used to obtain detailed, complete, and large amounts of data, for facilitating subsequent statistical processing and analysis [48].

Then, in data analysis, this study used methods such as multiple linear regression analysis. Firstly, the data characteristics are studied through descriptive statistics. Secondly, correlation analysis is used to determine the relationship between variables and whether there is collinearity, laying the foundation for multiple regression analysis. Furthermore, methods such as common method bias, reliability testing, and validity testing are used to determine the quality of the survey and the reliability of variable measurements. Finally, based on the theoretical model, multiple linear regression analysis was used to verify the causality between variables [48]. To verify the separate moderating and co-moderating effects of DC and IE, this study mainly adopts stepwise and bootstrap analysis methods to ensure the accuracy and effectiveness of regression results. The results of the stepwise method are highly significant and informative, but with low testing power [49]. However, the confidence interval obtained by the Bootstrap method is more accurate and has higher testing power, which is highly praised by scholars [50].

### 3.3 Research sample

According to the *Digital China Development Report 2020*, Zhejiang Province is the champion in industrial digitization index nationwide. The manufacturing industry has always been the powerhouse and foundation for local growth, with the new high-quality development “business card” of intelligent manufacturing. Shaoxing City vigorously promotes digital reforms with this as its goal. Therefore, our team conducted a survey in Shaoxing, Zhejiang Province from March 1, 2022, to April 30, 2022, using the questionnaire survey method. The survey was mainly conducted on-site, and the respondents provided a provided written informed consent. To fully answer research questions, this survey procedure is divided into three steps. Firstly, questionnaire design. The questionnaire was designed with a 6-point Likert scale based on existing literature and was reviewed by experts and corporate executives, which was finalized after multiple rounds of revisions. Secondly, determination of the research subjects. The research mainly focuses on all-type manufacturing enterprises in Shaoxing, Zhejiang Province, private or state-owned, domestic, or foreign-owned subjects, all covered in terms of general equipment, computer and communication and pharmaceuticals. Finally, research implementation. To collect samples as much as possible and ensure the questionnaire quality, supported by government departments of Shaoxing, questionnaires were distributed to middle and senior managers to ensure authentic responses. To realize higher quality, questionnaires without foreign business operations or the EDT were excluded. Finally, due to the limited resources and research capabilities required for enterprise research, a total of 203 valid questionnaires were collected. Cochran proposed a formula, which is $n_{o}=\frac{t^{2}\times s^{2}}{d^{2}}$ [51]. Based on the acceptable margin of error (d), t-value, and estimation of the standard deviation of the sample population (s), it was calculated that approximately 171 questionnaires were needed for this study. Therefore, 203 valid questionnaires met the needs of this study.

The other party uses descriptive statistical analysis to understand data characteristics and avoid outliers. In addition, common method bias testing and reliability and validity testing were used to determine the quality of the questionnaire and the reliability of the scale. This study was approved by the Ethics Committee of Department of Humanities and Social Sciences, Shaoxing University (No. 20210013).

### 3.4 Variable measurement

#### (1) Corporate internationalization

Referring to the studies by Schwens et al. [21] and Wang et al. [10], the measurement of corporate internationalization included two aspects. The breadth dimension was measured by the regions involved in the enterprise’s overseas sales in 2020, including North America, South America, Europe, Asia, Africa, and Oceania. The depth was measured by the proportion of cross-border e-commerce’s sales revenue to total sales revenue in 2020, ranging from 0% to 100% in intervals of 10%.

#### (2) EDT

There are four items to measure variable referring to the research by Li et al. [52] and others. Enterprise managers gave their answers accordingly that to what extent their enterprise currently used digital technologies in production activities, ranging from very little to very much. The specific technologies mentioned included laser cutting, waterjet cutting, 3D printing, along with tracking technologies like bar codes and radio frequency identification (RFID). Additionally, advanced manufacturing systems such as smart factory adaptive manufacturing and batch production were also included, together with automation processes involving automatic machine tools, handling equipment, and robots. Here, Cronbach’s Alpha coefficient for this variable is 0.865.

#### (3) DC

Based on previous centralization measurement by Jaworski and Kohli [22], this study modified it into four aspects that top management decision power regarding new projects, the adoption of new policies, the hiring of new employees, and employee promotions. Here, Cronbach’s Alpha coefficient for this variable is 0.926.

#### (4) IE

According to previous studies, the number of market entries, export ratio, and the duration of overseas operations can be used to measure IE. Specifically, the duration of overseas operations reflects the adaptability and knowledge accumulation in corporate internationalization. Therefore, following the study by Wu and Chen [28], this research measured IE by subtracting the year of the enterprise’s entry into international markets from 2021.

#### (5) Control variables

The control variables mainly include an enterprise’s operation length, measured by subtracting the year of its establishment from 2021; enterprise size, measured by the logarithm of the number of full-time employees; ownership type, measured by the enterprise’s ownership structure; industry type, measured by the primary business sector of the enterprise; foreign networks, referring to the studies by Cuevas-Rodrígue et al. [53], the measurement of foreign networks consists of three items: the establishment of new networking relationships with foreign entities, the formation of new cooperative relationships with foreign markets, and the development of personal relationships between executives and foreign clients. Here, the Cronbach’s Alpha coefficient for the foreign network variable is 0.911.

The main content of this questionnaire is as follows, as shown in Table 1.

**Table 1:** Questionnaire content.

Variables	Measurement content or method	Source
Corporate internationalization	The breadth dimension was measured by the regions involved in the enterprise’s overseas sales in 2020, including North America, South America, Europe, Asia, Africa, and Oceania. The depth was measured by the proportion of cross-border e-commerce’s sales revenue to total sales revenue in 2020, ranging from 0% to 100% in intervals of 10%.	Schwens et al. [21] and Wang et al. [10]
	The breadth dimension was measured by the regions involved in the enterprise’s overseas sales in 2020, including North America, South America, Europe, Asia, Africa, and Oceania. The depth was measured by the proportion of cross-border e-commerce’s sales revenue to total sales revenue in 2020, ranging from 0% to 100% in intervals of 10%.	
EDT	EDT1, laser cutting, waterjet cutting, 3D printing.	Li et al. [52]
	EDT2, tracking technologies like bar codes and radio frequency identification (RFID).	
	EDT3, advanced manufacturing systems such as smart factory adaptive manufacturing and batch production were also included	
	EDT4, automation processes involving automatic machine tools, handling equipment, and robots.	
	DC1, the decision of our company to launch new projects is always made by the top management.	
DC	DC2, the decision of our company to adopt new policies is always made by the top management.	Jaworski and Kohli [22]
	DC3, the decision of our company to hire new employees is always made by the top management.	
	DC4, the decision of our company’s employee promotions is always made by the top management.	
IE	This research measured IE by subtracting the year of the enterprise’s entry into international markets from 2021.	Wu and Chen [28]
Control variables	Enterprise’s operation length, measured by subtracting the year of its establishment from 2021;	Wang et al. [10], Wang et al. [11], Li et al. [52],
	Enterprise size, measured by the logarithm of the number of full-time employees.	
	Ownership type, measured by the enterprise’s ownership structure.	
	Industry type, measured by the primary business sector of the enterprise.	
	Foreign networks, the measurement of foreign networks consists of three items: FN1, the establishment of new networking relationships with foreign entities, FN2, the formation of new cooperative relationships with foreign markets. FN3, the development of personal relationships between executives and foreign clients.	Cuevas-Rodrígue et al. [53]

## 4. Empirical analysis

### 4.1 Descriptive statistics

The data in this study was analyzed by SPSS 26.0 and the Process 4.0 plugin. Tables 2 and 3 presents the descriptive statistics of the variables, including the mean, standard deviation, and correlations. To explore the relationships between various variables, understand whether there is a correlation between the independent variable, moderating variable, and dependent variable, together with verify research hypotheses and lay the foundation for subsequent multiple regression analysis, a correlation analysis was conducted. The results show that the EDT is significantly positively correlated with the breadth and depth of internationalization, significantly negatively correlated with the DC, and not correlated with IE. The DC is significantly negatively correlated with the breadth and depth of internationalization, and there is no correlation with IE. There is no significant correlation between IE and the breadth and depth of internationalization. The breadth and depth of internationalization are significantly correlated. The results are in line with expectations, which supports the hypothesis. The correlation coefficient between any variable is less than 0.4, and is less affected by multi-collinearity.

**Table 2:** Descriptive statistics.

Variable	N	Min	Max	Mean	Std. Dev.
Enterprise Operational Time	203	0	75	23.550	16.068
Enterprise Size	203	1.61	10.86	6.110	1.767
Ownership Type	203	0	1	0.722	0.450
Industry Type	203	0	1	0.219	0.415
Foreign Networks	203	1	6	3.417	1.282
EDT	203	1	6	3.088	1.460
DC	203	1	6	3.570	1.383
IE	203	0	7.61	2.630	0.977
Internationalization Breadth	203	1	6	3.530	1.754
Internationalization Depth	203	0	10	1.810	1.905

**Table 3:** Variable correlations.

	1	2	3	4	5	6	7	8	9	10
1. Enterprise Operational Time	1									
2. Enterprise Size	0.329^**^	1								
3. Ownership Type	0.002	0.095	1							
4. Industry Type	-0.180^*^	-0.262^**^	-0.065	1						
5. Foreign Networks	0.014	-0.014	0.145	-0.043	1					
6. EDT	0.123	0.391^**^	-0.054	-0.238^**^	-0.044	1				
7. DC	-0.205^*^	-0.087	0.034	-0.038	0.376^**^	-0.169^*^	1			
8. IE	0.280^**^	0.024	-0.149	0.032	-0.12	0.002	-0.032	1		
9. Internationalization Breadth	0.057	0.267^**^	-0.074	-0.133	-0.018	0.335^**^	-0.164^*^	0.158	1	
10. Internationalization Depth	0.031	-0.011	0.103	-0.007	-0.083	0.168^*^	-0.186^*^	0.041	0.198^*^	1

(Note: ** indicates p < 0.01

* indicates p < 0.05.)

### 4.2 Reliability and validity testing

For common method bias, this study used the Haman single factor method for testing. The results showed that the proportion of the first factor variation in the total variation was 28.685%, less than 40%, indicating that there was no single factor explaining most of the variation. Exploratory factor analysis indicates that, the KMO values for the EDT, the DC and foreign networks are both above 0.7, and Bartlett’s sphericity test is significant, indicating that each variable has good construct validity. The Cronbach’s Alpha of all variables is greater than 0.86, and the combined reliability (CR) is greater than 0.9, indicating high internal consistency and good reliability among the variables. The factor loading of all variables are greater than 0.7, and the average extraction variance (AVE) is greater than 0.7, indicating that each variable has good convergent validity, as shown in Table 4.

**Table 4:** Cronbach’s alpha, combination reliability, and average variance extraction.

Variables	Items	Factor 1	Factor 2	Factor 3	Cronbach’s Alpha	AVE	CR
EDT	EDT1		0.779		0.865	0.715	0.909
	EDT2		0.832				
	EDT4		0.852				
	EDT3		0.902				
DC	DC3	0.853			0.926	0.819	0.947
	DC2	0.871					
	DC1	0.901					
	DC4	0.912					
Foreign Networks (FN)	SC3			0.871	0.911	0.850	0.944
	SC2			0.913			
	SC1			0.922			

### 4.3 Hypothesis testing

#### 4.3.1 Testing of major effects and moderating effects

Firstly, the theoretical model was examined by multiple regression analysis, and analysis was conducted on EDT influence on the breadth and depth of internationalization, as well as the moderating effect of DC and IE. The results are shown in Tables 5 and 6. According to Models 2 and 8, EDT’s influence on internationalization breadth (β = 0.258, p < 0.01) and depth (β = 0.156, p < 0.1) is both significantly positive. The EDT helps identify international market opportunities and develop international markets, thereby enhancing internationalization breadth, so that H1 and H2 are confirmed. To test the moderating effect of DC, centering was conducted on EDT and DC to solve collinearity. As a result, Models 3 and 4 indicate that DC has a significant negative influence on internationalization breadth (β = -0.145, p < 0.1), while IE has an opposite effect (β = 0.188, p < 0.05). This indicates that DC is not conducive to enhancing the breadth of internationalization of enterprises, while IE is the opposite. Furthermore, Models 5 and 6 demonstrate that DC negatively moderates the relationship between EDT and internationalization breadth (β = -0.411, p < 0.01), whereas IE’s corresponding effect is positive (β = 0.712, p < 0.01). This suggests that low DC is more conducive to improving decision-making quality, stimulating employee initiative, promoting cross departmental cooperation, and thus stimulating the positive influence of EDT on the internationalization breadth. And high IE is more conducive to identifying opportunities and risks, overcoming the disadvantages of outsiders, alleviating cost pressures, and stimulating the positive influence of EDT on the internationalization breadth. Therefore, H3 and H5 are verified.

**Table 5:** Results of multiple linear regression analysis (Dependent variable: Internationalization breadth).

Variables	Model 1	Model 2	Model 3	Model 4	Model 5	Model 6
Enterprise Operational Time	-0.047	-0.04	-0.072	-0.133	-0.129	-0.146
Enterprise Size	0.273^**^	0.178^*^	0.183^*^	0.192^*^	0.213^*^	0.22^**^
Ownership Type	-0.105	-0.08	-0.086	-0.062	-0.071	-0.066
Industry Type	-0.077	-0.037	-0.05	-0.063	-0.056	-0.066
Foreign Networks	-0.002	0.006	0.06	0.086	0.128	0.118
EDT		0.258^**^	0.234^**^	0.234^**^	0.544^***^	-0.077
DC			-0.145+	-0.161+	0.043	0.05
IE				0.188^*^	0.197^*^	-0.049
EDT^*^DC					-0.411^*^^*^	-0.44^**^
EDT^*^IE						0.712^**^
F	2.804^*^	3.985^***^	3.858^***^	4.195^***^	4.731^***^	5.115^***^
R2	0.088	0.142	0.159	0.19	0.232	0.268
△R2	0.088	0.054	0.016	0.031	0.042	0.036

(Note: EDT represents enterprise digital technology, DC represents decision concentration, and IE represents IE, the same below.

^***^ indicates p < 0.001

^**^ indicates p < 0.01, and

^*^ indicates p < 0.05.)

**Table 6:** Results of multiple linear regression analysis (Dependent variable: Internationalization depth).

Variables	Model 7	Model 8	Model 9	Model 10	Model 11	Model 12
Enterprise Operational Time	0.064	0.066	0.021	-0.008	-0.005	0.001
Enterprise Size	-0.013	-0.068	-0.068	-0.066	-0.059	-0.053
Ownership Type	-0.145	-0.127	-0.124	-0.141	-0.15	-0.164^*^
Industry Type	-0.053	-0.063	-0.079	-0.071	-0.072	-0.079
Foreign Networks	-0.095	-0.087	-0.026	-0.016	0.005	0.007
EDT		0.156+	0.134	0.137	0.144+	0.501+
DC			-0.191^*^	-0.193^*^	-0.192^*^	-0.195^*^
IE				0.096	0.097	0.091
EDT^*^DC					-0.153+	-0.159^*^
EDT^*^IE						-0.379
F	0.991	1.396	1.931+	1.854+	2.101^*^	2.125^*^
R2	0.031	0.052	0.082	0.090	0.113	0.126
△R2	0.031	0.021	0.030	0.008	0.023	0.013

Note: *** indicates p < 0.001

** indicates p < 0.01

* indicates p < 0.05, and + indicates p < 0.1.

Similarly, Models 9 and 10 reveal that DC has a significant negative influence on internationalization depth (β = -0.191, p < 0.05), while IE doesn’t. Additionally, Model 11 shows that DC negatively moderates the relationship between EDT and internationalization depth (β = -0.159, p < 0.05). It indicates that low DC is more conducive to improving decision-making quality, stimulating employee initiative, promoting cross departmental cooperation, and thus stimulating the positive influence of EDT on the internationalization dept. However, there is no significant such moderating effect by IE. The possible reason is that IE can be applied to the expansion of similar national businesses, investments, etc., promoting the internationalization breadth. However, business expansion in the same country requires more resources, capabilities, and costs, which limits the effectiveness of EDT [15, 16]. At the same time, cultural differences and other factors face a higher disadvantage for outsiders [14]. In conclusion, H4 is supported, but H6 is not supported.

#### 4.3.2 Testing of co-moderating effect

In order to analyze the co-moderating effect of DC and IE on the relationship between EDT and internationalization breadth (internationalization depth), and to verify H7 and H8, this study employed M1 and M2 models in PROCESS4.0 through the Bootstrap method, as shown in Tables 7–9. In Table 7, Model 13 indicates that the interaction between EDT and DC has a significant negative influence on internationalization breadth (β = -0.179, p < 0.01), while the interaction between EDT and IE has a significant positive effect (β = 0.296, p < 0.01). Similarly, Model 14 demonstrates that the interaction between EDT and DC negatively influences internationalization depth (β = -0.135, p < 0.05), while the interaction between EDT and IE has no significant influence on internationalization depth. Therefore, these findings further validate H3, H4, and H5.

**Table 7:** The results of the regression analysis on the joint moderating effects of DC and IE on internationalization breadth (depth).

Variables	Internationalization breadth (model 13)			Internationalization depth (model 14)		
	β	SE	t	β	SE	t
Constant	2.197^**^	0.657	3.342	2.074^**^	0.674	3.076
EDT	0.295^**^	0.098	3.019	0.175	0.109	1.601
DC	-0.227^*^	0.104	-2.185	-0.264^*^	0.115	-2.299
IE	0.394^**^	0.140	2.820	0.172	0.158	1.089
EDT^*^DC	-0.179^**^	0.059	-3.025	-0.135^*^	0.067	-2.027
EDT^*^IE	0.296^**^	0.113	2.611	-0.186	0.126	-1.480
Enterprise Operational Time	-0.016	0.009	-1.747	0.000	0.011	0.014
Enterprise Size	0.219	0.084	2.604	-0.054	0.091	-0.602
Ownership Type	-0.257	0.292	-0.880	-1.332	0.656^*^	-2.032
Industry Type	-0.278	0.327	-0.852	-1.092	1.082	-1.010
Foreign Networks	0.162	0.111	1.455	0.010	0.122	0.082
F	5.115^***^			2.125^*^		
R2	0.268			0.126		

Note: *** indicates p < 0.001

** indicates p < 0.01

* indicates p < 0.05, and + indicates p < 0.1.

**Table 8:** Effect of EDT on internationalization breadth under different DC and IE level.

DC	IE	Effect	se	t	p	LLCI	ULCI
-1.383	-0.977	0.253	0.164	1.538	0.126	-0.072	0.577
-1.383	0.000	0.542	0.129	4.202	0.000	0.287	0.796
-1.383	0.977	0.831	0.175	4.738	0.000	0.484	1.177
0.000	-0.977	0.006	0.146	0.038	0.970	-0.283	0.294
0.000	0.000	0.295	0.098	3.019	0.003	0.102	0.487
0.000	0.977	0.584	0.149	3.909	0.000	0.288	0.879
1.383	-0.977	-0.241	0.170	-1.421	0.158	-0.577	0.094
1.383	0.000	0.048	0.126	0.379	0.706	-0.201	0.296
1.383	0.977	0.337	0.165	2.041	0.043	0.011	0.662

**Table 9:** The results of the influence of EDT on internationalization depth across different levels of DC and IE.

DC	IE	Effect	se	t	p	LLCI	ULCI
-1.393	-0.998	0.548	0.190	2.884	0.005	0.173	0.924
-1.393	0.000	0.363	0.146	2.482	0.014	0.074	0.651
-1.393	0.998	0.177	0.195	0.907	0.366	-0.208	0.562
0.000	-0.998	0.361	0.160	2.254	0.026	0.045	0.677
0.000	0.000	0.175	0.109	1.601	0.112	-0.041	0.391
0.000	0.998	-0.011	0.173	-0.062	0.951	-0.352	0.330
1.393	-0.998	0.173	0.179	0.965	0.336	-0.182	0.528
1.393	0.000	-0.013	0.140	-0.090	0.929	-0.290	0.265
1.393	0.998	-0.198	0.197	-1.008	0.315	-0.587	0.190

Table 8 demonstrates that EDT’s influence on Internationalization breadth varies by different levels of DC and IE. When DC is low and IE is high, the confidence interval is [0.484, 1.177] excluding zero, indicating significant effects (β = 0.831, p < 0.001). Similarly, when both DC and IE are high, the confidence interval is [0.011, 0.662] excluding zero, also showing significant effects (β = 0.337, p < 0.05). However, when both DC and IE are low, the confidence intervals include zero, indicating no significant effect. All these findings show that EDT’s influence differs significantly according to different levels of DC and IE. Specifically, when DC is low and IE is high, or when both DC and IE are high, they strengthen the positive influence of EDT on internationalization breadth, with the former having a stronger effect. It indicates that when expanding the international market, decision-making speed can be improved under certain conditions such as high IE and high DC, to respond promptly to changes in the external environment and have a positive influence on enterprises. Therefore, enterprises cannot blindly deny the DC. Meanwhile, this also confirms the DC and IE jointly moderate the relationship between EDT and internationalization breadth, thus supporting H7.

According to Table 9, EDT’s influence on internationalization depth also varies by different levels of DC and IE. When both DC and IE are low, the confidence interval is [0.173, 0.924], excluding zero, indicating significant effect (β = 0.548, p < 0.01). Similarly, with low DC and moderate IE, the confidence interval is [0.074, 0.651] excluding zero, indicating significant effect (β = 0.363, p < 0.05). However, for the other three combinations, namely low DC and high IE, or high DC with and low or high IE, the confidence intervals all include zero, indicating no significance effects. As a result, significant differences in the influence of EDT greatly by different levels of DC and IE. Specifically, both low DC and low IE can strengthen EDT’s positive influence on internationalization depth, which indicates that DC and IE jointly moderate the relationship between EDT and internationalization depth, thus supporting H8. The reason is that IE can be categorized by breadth and depth. That is, the more extensive the IE can lower enterprises’ sensitivity to uncertain environmental changes. And more IE may weaken an enterprise’s in-depth thinking and adaptability to a particular region with lower decision accuracy as it will overestimate potential success of investment in countries with similar environments, finally resulting in investment failures. In addition, IE eventually stabilizes as it deepens, leading to diminishing marginal effects of its moderating role on internationalization depth [44].

**Fig 2:**
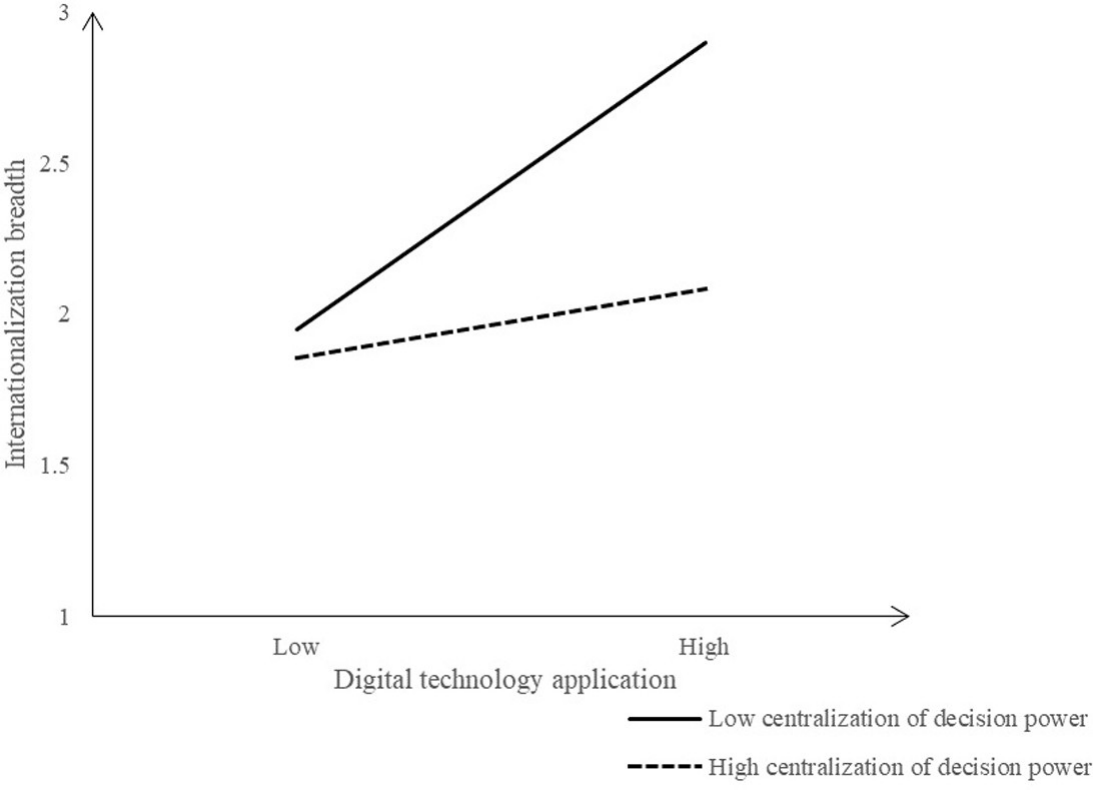
Moderating effect of DC (Dependent variable: Internationalization breadth).

**Fig 3:**
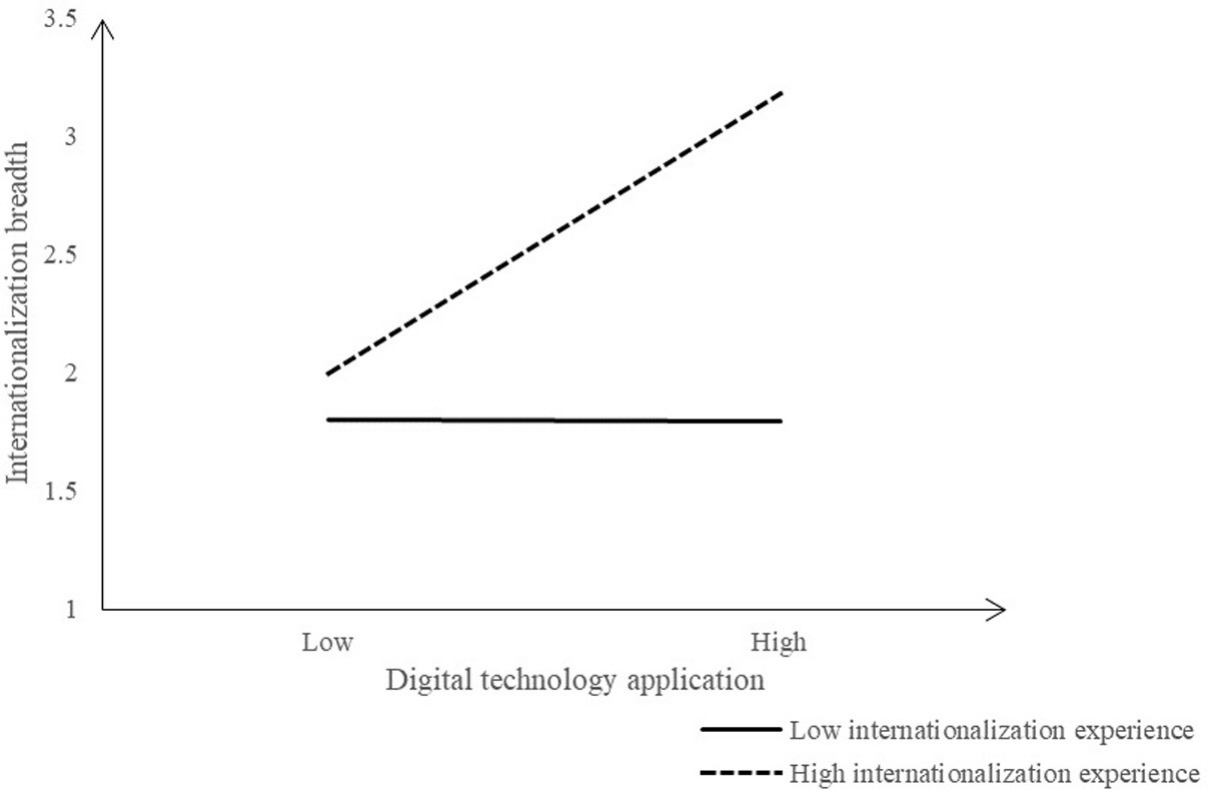
Moderating effect of IE (Dependent variable: Internationalization breadth).

**Fig 4:**
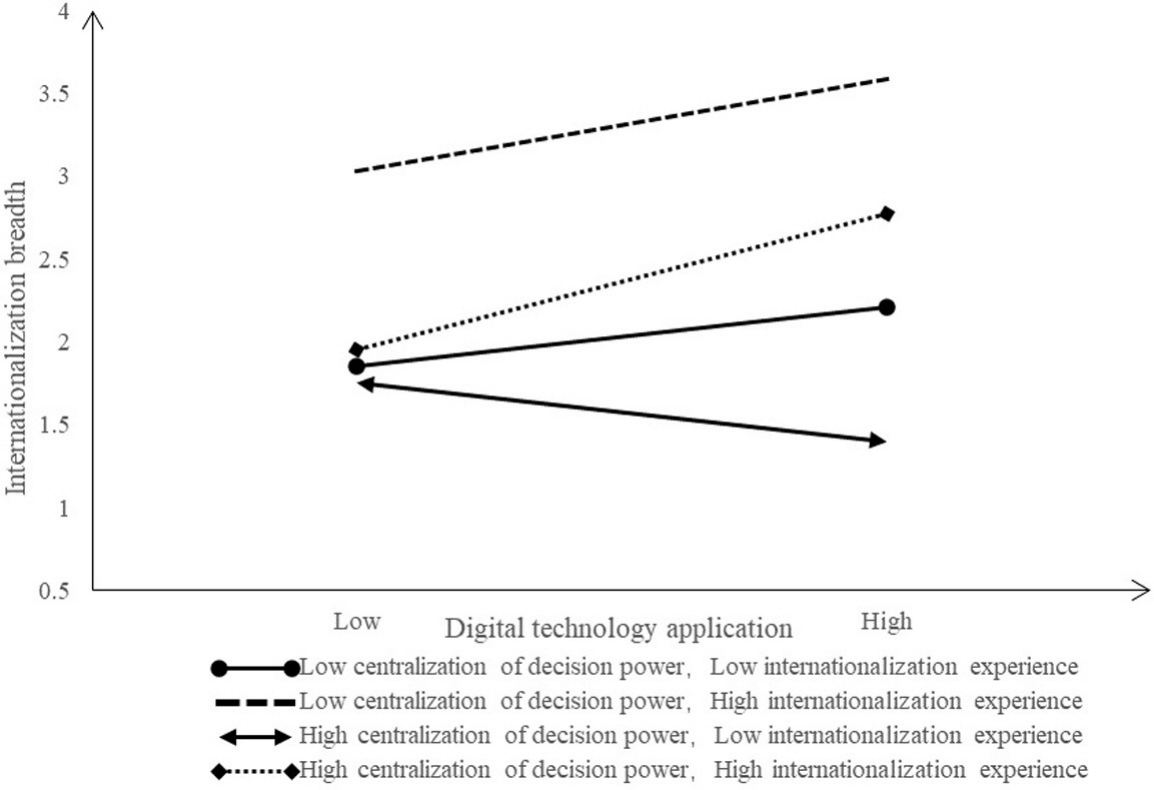
Joint moderating effect (Dependent variable: Internationalization breadth).

**Fig 5:**
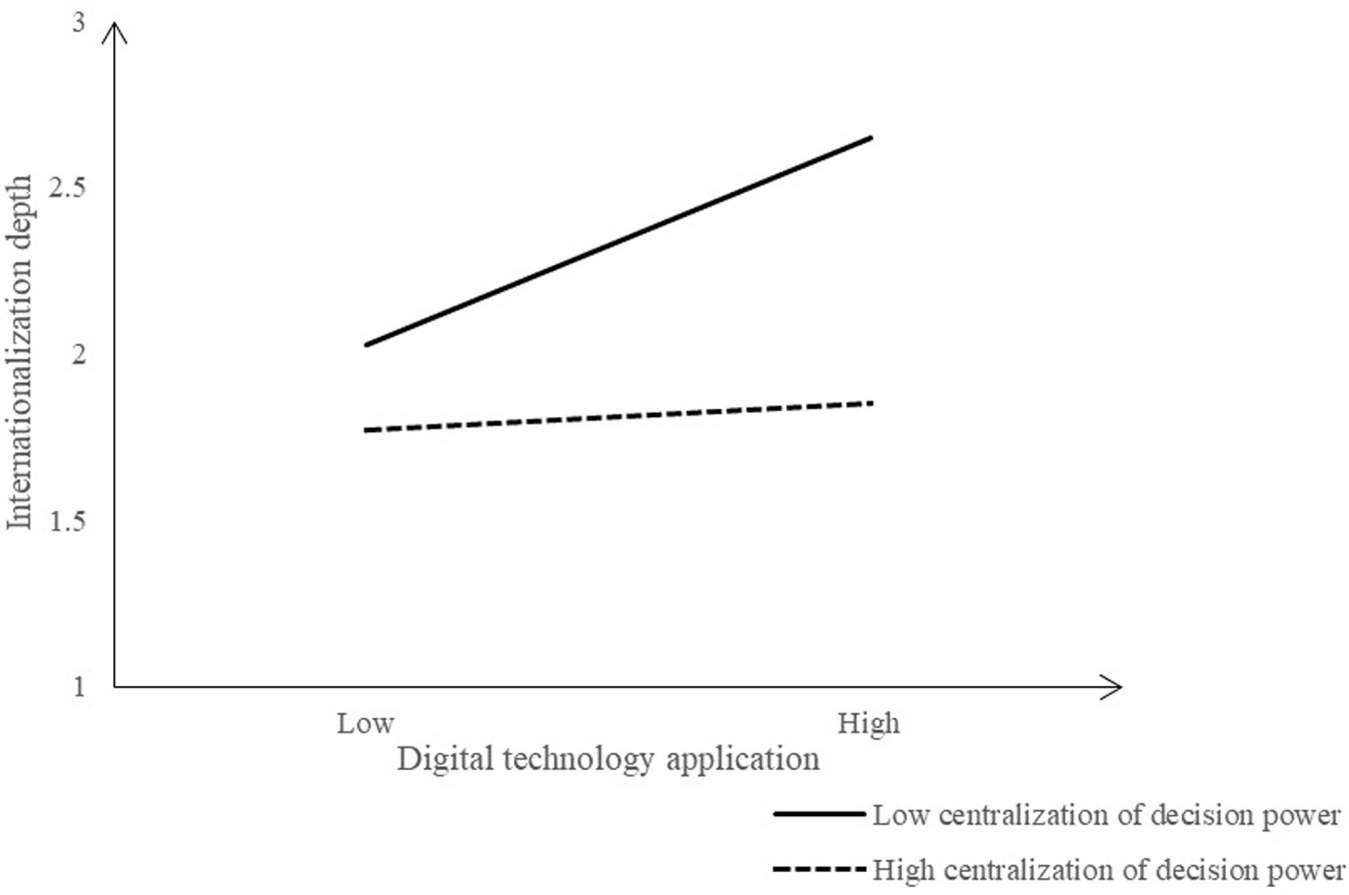
Moderating effect of DC (Dependent variable: Internationalization depth).

**Fig 6:**
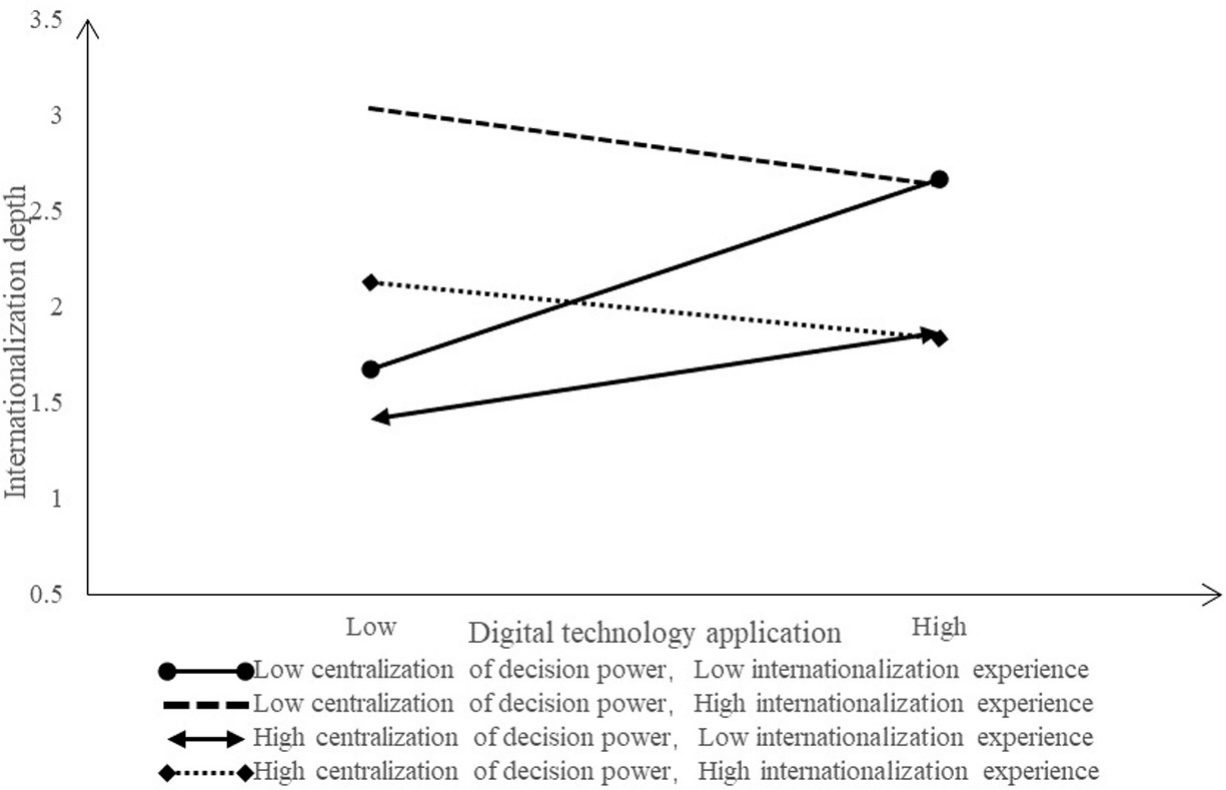
Joint moderating effect (Dependent variable: Internationalization depth).

To vividly present the separate moderating and co-moderating effects of DC and IE, this study utilized regression coefficients to illustrate the variations in corporate internationalization at different levels of DC and IE. For details, please refer to Figs 2 to 6. The Fig 2 shows the moderating effect of DC between EDT and internationalization breadth. When the DC is low, the positive influence of EDT on the internationalization breadth increases significantly, but higher DC leads to less positive influence. That is, lower DC is better to stimulate the positive influence of EDT on the internationalization breadth.

The Fig 3 shows the moderating effect of IE between EDT and internationalization breadth. When the IE is low, the positive influence of EDT on the internationalization breadth increases less and shows a downward trend; When there is a high level of IE, the positive influence of EDT on internationalization breadth increases significantly. That is, higher IE can contribute to stimulating the positive influence of EDT on internationalization breadth.

The Fig 4 shows the co-moderating effect of DC and IE between EDT and internationalization breadth. When the DC is low and the IE is high, as well as when the DC is high and the IE is high, the positive influence of EDT on internationalization breadth increases significantly, which is conducive to strengthening the positive influence of EDT on internationalization breadth. In other cases, the positive influence of EDT on the internationalization breadth has increased slightly or even decreased.

Similarly, the Fig 5 shows the moderating effect of DC between EDT and internationalization depth. When the DC is low, the positive influence of EDT on the internationalization depth increases significantly; when the DC is high, the positive influence of EDT on the internationalization depth increases less. That is, higher DC will weaken the positive influence of EDT on the internationalization depth.

The Fig 6 shows the co-moderating effect of DC and IE between EDT and internationalization depth. When DC is low and IE is low, the positive influence of EDT on internationalization depth increases significantly, which is more conducive to strengthening the positive influence of EDT on internationalization depth. In other cases, the positive influence of EDT on the internationalization depth has increased slightly or even decreased.

## 5. Conclusions and implications

### 5.1 Conclusions

EDT has grown into a new driver for higher level of corporate internationalization, but there are few existing research on relevant topics, especially underlying mechanisms. Among 203 valid questionnaires gathered in this study, manufacturing enterprises in Zhejiang Province are taken as targets. Through analysis, the results are as follows: (1) EDT positively influences internationalization breadth significantly. (2) DC and IE respectively moderate the relationship between EDT and internationalization breadth. (3) DC and IE play a co-moderate role between the EDT and the internationalization breadth, that is, there are significant differences in EDT’s influence on internationalization breadth at different levels of DC and IE. Specifically, low DC and high IE, or high DC and high IE are beneficial to strengthen the positive influence of EDT on internationalization breadth, with the former having a stronger effect. (4) Similarly, EDT positively influences internationalization depth significantly. (5) DC also moderates the relationship between EDT and internationalization depth. (6) DC and IE play a co-moderate role between the EDT and the internationalization depth, that is, EDT’s influence on internationalization depth is difference at different levels of DC and IE. Particularly, both low DC and low IE are favorable to enhance the positive influence of EDT on internationalization depth.

The results of this study are like those conducted by Adomako et al., Wang et al., Wang et al., Li et al. and Li et al. [9–13], all of which confirm that the EDT has a positive influence on the enterprises internationalization. Unlike existing studies, the types of internationalization are distinguished, then the influence of EDT on the of internationalization breadth and depth are analyzed. The former has a stronger effect, that is, the EDT is more conducive to the reuse of resources and capabilities by enterprises, expanding to multiple countries at a lower cost, and realizing the expansion of enterprise internationalization breadth. But when further expanding to the same country, the competition pressure is greater, which will be constrained by resources and capabilities, reaching a saturation stage. At the same time, this study expanded the context considerations of the influence of EDT on enterprise internationalization and found the joint effect of DC and IE. When enterprises enhance the internationalization breadth and depth, they need to consider different decision-making methods and the amount of experience. That is, when expanding the internationalization breadth, there are two potential strategies: low DC and high IE or high DC and high IE. When considering internationalization depth, low DC and low IE strategy is necessary.

### 5.2 Implications

Firstly, enterprises’ engagement in internationalization activities should strengthen the EDT. Whether it is promoting enterprise expansion to multiple countries or achieving deep extension within a country, digital technology plays a positive role. Therefore, it is recommended that enterprises utilize technologies such as big data and artificial intelligence in their operational management, production, and transportation. It will result in better domestic and international interactions, higher flexibility, agility, and adaptability, along with less organizational and situational constraints in response to the complex and ever-changing international environment. In addition, enterprises should leverage digital technologies to enhance their capabilities in data acquisition, analysis, and utilization during international expansion, to swiftly update products or services, and timely respond to the personalized, diverse, and evolving needs of consumers by evaluating and predicting consumption trends in different countries or regions.

Secondly, it is important to avoid excessive DC. Moderate DC facilitates faster decision-making, quicker response to international markets, and higher resource allocation efficiency for R & D and innovations. Moreover, considering co-moderating effect of DC and IE, when expanding the o internationalization breadth for enterprises, based on their abundant experience in internationalization, enterprises can consider both moderate DC and IE to facilitate the development of their internationalization endeavors. And reasonable separation of powers can also obtain more information and knowledge, thereby realizing higher quality and accuracy of decision-making. But in order to enhance the internationalization depth, when there is a lack of IE, it is best to decentralize power appropriately to obtain more information, stimulate employee enthusiasm, enhance cooperation efficiency, and improve the quality and accuracy of decision-making.

The last point is to enhance IE. Enterprises need to learn from global benchmarks and analyze their own issues faced during overseas operations. In that way, they can draw lessons from previous success stories and failures, to enhance their perception of risks, along with greater internationalization depth and breadth. However, it should be recognized that IE is not omnipotent. When extending to a same country, it is necessary to face higher competitive pressure and the constraints of the company’s own resources and capabilities.

### 5.3 Limitations and future directions

Despite insights provided by this study, there are still some limitations to be further solved in future studies. Firstly, this study primarily focuses on contextual conditions rather than specific pathways that EDT influences corporate internationalization, which is not conducive to revealing the black box within it. So future studies could delve into the mediating mechanisms and processes to better understand the underlying dynamics. Secondly, the types of internationalization of enterprises were not fully distinguished, such as exports, mergers and acquisitions, joint ventures, etc. Therefore, the next step can be done to provide a more comprehensive understanding of how digital technology applications affect the breadth and depth of internationalization of different types.
